# Development of a multi-year pediatric antibiogram in Georgia identifies antibiotic resistance changes over the past ten years

**DOI:** 10.1017/ash.2025.32

**Published:** 2025-02-12

**Authors:** Matthew Linam, Madeleine Goldstein, Tracy Huang, Adrianna Westbrook, Robert C. Jerris, Mark D. Gonzalez

**Affiliations:** 1 Division of Pediatric Infectious Diseases, Emory University School of Medicine, Atlanta, GA, USA; 2 Children’s Healthcare of Atlanta, Atlanta, GA, USA; 3 Pediatric Biostatistics Core, Department of Pediatrics, Emory University, Atlanta, GA, USA; 4 Department of Pathology and Laboratory Medicine, Emory University School of Medicine, Atlanta, GA, USA

## Abstract

**Background:**

Antibiograms monitor antibiotic resistance trends and help guide empiric antibiotic treatment. A statewide pediatric antibiogram can help inform stewardship efforts.

**Methods:**

Annual pediatric antibiograms for the five children’s hospitals in Georgia from 2014–2023 were collected. All sites used the Clinical and Laboratory Standards Institute guidelines for antibiogram development. Antibiogram data were combined, and the most common bacteria were included: Staphylococcus aureus, Streptococcus pneumoniae, Enterococcus faecalis, Escherichia coli, Klebsiella pneumoniae, Enterobacter cloacae complex and Pseudomonas aeruginosa. Interhospital differences were compared for methicillin-susceptible S. aureus (MSSA), methicillin-resistant S. aureus (MRSA), E. coli and K. pneumoniae. The combined data from 2014 and 2023 were compared to demonstrate antibiotic susceptibility changes over time.

**Results:**

Data in 2023 for MSSA and MRSA showed clindamycin susceptibility was 78% and 82%, respectively. S. pneumoniae susceptibility to amoxicillin/clavulanate was 96%. E. faecalis resistance to ampicillin and vancomycin was rare. For all included gram-negative bacteria, susceptibility remained high to 3^rd^ generation cephalosporins (90%–92%) and meropenem (95%–99%). From 2014 to 2023, the rate of MRSA decreased from 49% to 33.5%. S. pneumoniae susceptibility to amoxicillin/clavulanate and clindamycin significantly increased. For E. coli, there was a significant decrease in susceptibility for cefazolin (90% to 84%), ceftriaxone (95% to 92%), and meropenem (100% to 99%). There were nonsignificant decreases in susceptibility for K. pneumoniae.

**Conclusion:**

Over the past 10 years, MRSA rates decreased, S. pneumoniae antibiotic susceptibility increased, and gram-negative bacilli susceptibility was stable to slightly decreased. Georgia antibiogram data support the recommended antibiotic treatment for common pediatric infections.

Antibiograms provide cumulative antibiotic susceptibility for specific pathogens and can be used to show antibiotic resistance trends over time. They are recommended by the Centers for Disease Control and Prevention and the Infectious Diseases Society of America as an important component of antibiotic stewardship programs.^1,2^ Antibiogram data can be incorporated into local treatment guidelines and clinical pathways informing empiric treatment recommendations for infections such as urinary tract infections, skin and soft tissue infections, and community-acquired pneumonia. Access to antibiogram data can also shorten the time to appropriate antibiotics by optimizing the initial antibiotic chosen to treat an infection.^3,4^

The Clinical and Laboratory Standards Institute (CLSI) M39 guideline outlines the recommendations for the development of antibiograms in healthcare settings.^5^ To improve accuracy of the antibiotic susceptibility data, the guideline recommends only pathogens with at least 30 unique isolates should be included in the cumulative antibiogram. While healthcare facilities can combine multiple years of data to achieve these denominators, this still presents challenges for small facilities.^6^ Large free-standing children’s hospitals are typically able to meet the isolate recommendations for multiple bacteria, but smaller children’s hospitals or those connected to an adult facility may only meet these thresholds for the most commonly isolated pathogens such as *Staphylococcus aureus* or *Escherichia coli*.

To overcome these challenges, antibiograms from multiple facilities have been combined to create statewide or regional antibiograms.^6–10^ The majority of these combined geographic antibiograms are from adult facilities, whose resistance patterns may not accurately represent resistance patterns in children. There are several challenges in combining antibiogram data from multiple facilities. Although there are standard breakpoints recommended in the CLSI guidelines, adoption of updated breakpoints may be delayed.^4^ This creates inaccuracies when trying to aggregate antibiotic susceptibility data from facilities using different breakpoints. When combining isolates from different-sized facilities, the number of isolates included from larger, tertiary, children’s hospitals may overrepresent the data being combined into a regional antibiogram skewing results towards greater antibiotic resistance.^11,12^ Despite the challenges of creating regional or statewide antibiograms, there is a need to provide antibiotic susceptibility data and temporal resistance trends to pediatric clinicians in smaller facilities and those practicing in the community.

This project was a collaboration between the five children’s hospitals in Georgia with the goal of creating the first combined pediatric antibiogram for the state of Georgia. We aimed to determine whether a combined antibiogram accurately represented the antibiotic susceptibility patterns reported from different parts of the state. Finally, we endeavored to describe the antibiotic susceptibility patterns for common bacteria and identify changes in resistance over time.

## Methods

A project team consisting of the director and associate director of microbiology and pediatric infectious diseases physicians from Children’s Healthcare of Atlanta oversaw the development of the statewide pediatric antibiogram. The project was reviewed by the Children’s Healthcare of Atlanta Institutional Review Board and determined to not be human subjects research. The five children’s hospitals in Georgia: Children’s Healthcare of Atlanta (Atlanta), Children’s Hospital of Georgia (Augusta), Beverly Knight Olsen Children’s Hospital (Macon), The Children’s Hospital (Columbus) and Dwaine & Cynthia Willett Children’s Hospital of Savannah (Savannah) were contacted and agreed to share their annual pediatric-specific antibiograms. A brief questionnaire was sent to hospital leadership and the person responsible for antibiogram development at each hospital. Questions focused on characteristics related to number and type of pediatric-specific beds, admissions, different patient populations cared for at the hospital, the patient population included in the pediatric antibiogram, who was responsible for overseeing the antibiogram development, and whether current CLSI guidelines and breakpoints were followed. Children’s Healthcare of Atlanta represents a healthcare system and includes data from Egleston and Scottish Rite Children’s Hospitals, and a portion of urgent care patients.

The annual pediatric antibiograms from each hospital from 2014 through 2023 were provided. After reviewing the antibiograms from each hospital, the pathogens that were consistently included in each hospital’s antibiogram were included: *S. aureus, Streptococcus pneumoniae, Enterococcus faecalis, E. coli, Klebsiella pneumoniae, Enterobacter cloacae* complex and *Pseudomonas aeruginosa*. Methicillin-susceptible *S. aureus* (MSSA) and methicillin-resistant *S. aureus* (MRSA) susceptibility data were reported separately. The pathogen-antibiotic combinations included for each bacterium were chosen if data were reported by at least three of the five hospitals and are shown in the Supplemental Table.

Each hospital antibiogram reported data as percent susceptible and the total number of isolates for each pathogen-antibiotic combination. The total number of isolates (denominator) and percent susceptible data were used to calculate the number of susceptible isolates (numerator). The numerator and denominator data for each pathogen-antibiotic combination from the individual hospital antibiograms were combined to create the statewide antibiogram. For hospitals that created distinct antibiograms for specific groups (e, pediatric intensive care or neonatal units) or by source (eg, urine), all available antibiogram data were combined to create a composite antibiogram. All isolates were included in the statewide antibiogram, even if fewer than 30 isolates were contributed from the individual hospital. Antibiogram data (percent susceptible and total isolates for each pathogen-antibiotic combination) were entered into an electronic tool. For 2014–2019, the project team entered the antibiogram data directly from each hospital’s published antibiograms. For 2020–2023, antibiogram data were collected prospectively once that year’s antibiogram was completed. Hospitals generated an antibiogram report that was not separated by hospital location or source, and these data were entered into the electronic tool. The project team reviewed data from each hospital for errors or inconsistencies and these were clarified or corrected prior to adding the data to the statewide antibiogram.

Antibiotic susceptibility rates for 2023 for Georgia (statewide data) and the five hospitals were compared to understand differences between individual hospital antibiogram data and the statewide antibiogram data. To limit differences that could be due to small sample sizes at individual hospitals, the analysis focused on four bacteria for which the hospitals had at least 30 isolates: MSSA, MRSA, *E. coli,* and *K. pneumoniae*. The exception was Columbus, which had fewer than 30 isolates for MRSA and *K. pneumoniae*. The pathogen-antibiotic combinations included in the comparison represented different antibiotic classes (i.e. beta-lactam and non-beta-lactam antibiotics). Antibiotics, such as vancomycin and meropenem, were omitted from the interhospital analysis if susceptibility was consistently 99%–100% across all locations. For each pathogen-antibiotic combination, forest plots showing the antibiotic susceptibility rate and 95% confidence interval (CI) were compared for Georgia and the individual hospitals.

Antibiotic susceptibility data for the five hospitals were combined to show changes in susceptibility from 2014 to 2023. Pathogen-antibiotic combinations included in the analysis for each bacterium represented different classes of antibiotics. Changes in percent susceptibility for 2014 versus 2023 were compared using the two-proportion z-test with Yates continuity correction, and all tests were two-sided. Adoption of the CLSI uncomplicated urinary tract infections (UTI) breakpoints for cefazolin (≤16 µg/mL) for *E. coli* began in 2017; therefore, 2017 and 2023 susceptibility data were compared. The Mann-Kendall test was used to evaluate trends over time from 2014 through 2023 for the MRSA rate. All analyses were performed with SAS 9.4 (Cary, NC) and R v4.4.0 (Vienna, Austria). A p-value below 0.05 was considered significant.

## Results

The hospital characteristics and antibiogram information are described in Table 1. Although all hospitals used the CLSI guidelines for antibiogram development, there were notable differences. Hospitals only included the first isolate of an organism for a specific patient for a given calendar year except for Augusta and Columbus, which may have more than one isolate for a specific patient for a given year. Augusta did not adjust for inducible resistance for *S. pneumoniae* or *S. aureus*. Hospitals adopted the updated CLSI breakpoints at different times for carbapenems (2012–2020), cephalosporins (2017–2020), and fluoroquinolones (2019–2022). Because Atlanta had not yet adopted the updated fluoroquinolone breakpoints, their fluoroquinolone data were not included starting in 2021, decreasing the number of isolates included for fluoroquinolones.

**Table 1.** Descriptive characteristics of the children’s hospitals participating in the development of a Georgia pediatric antibiogram


	**Children’s Healthcare** **of Atlanta**	**Children’s Hospital** **of Georgia**	**Beverly Knight Olson Children’s Hospital**	**The Children’s Hospital, Piedmont Columbus Regional**	**Dwaine & Cynthia Willett** **Children’s Hospital of Savannah**
**Location**	Atlanta, GA	Augusta, GA	Macon, GA	Columbus, GA	Savannah, GA
**Children’s hospital characteristics**					
Free-standing children’s hospital	Yes	Yes	No	No	No
Annual number of pediatric inpatient admissions	27,760	3,300	2,947	983	3,595
Annual number of pediatric emergency department visits	218,000	39,000	21,595	36,000	43,338
Annual number of pediatric urgent care visits	153,462	0	18,520	0	0
Annual number of pediatric outpatient visits	1,057,281	80,500	39,563	12,316	30,000
Total number of pediatric beds	673	149	110	24	36
Total number of pediatric ICU beds	113	14	21	5	14
Total number of neonatal ICU beds	89	45	66	46	92
Provides care for pediatric oncology patients	Yes	Yes	Yes	Yes	Yes
Provides pediatric blood and marrow transplantation	Yes	No	No	No	No
Provides pediatric organ transplantation	Yes	Yes	No	No	No
**Microbiology lab characteristics**					
Antibiogram developed using CLSI M39 guidelines	Yes	Yes	Yes	Yes	Yes
Primary methodology used for susceptibility testing	Microscan	Vitek2	Vitek2	Microscan	Microscan
Responsible for preparing the antibiogram					
Microbiology	Yes	Yes	Yes	No	No
Pharmacy	No	Yes	Yes	Yes	Yes
Hospital epidemiologist/infection prevention	No	Yes	No	No	No
Infectious diseases	No	Yes	No	No	No
Other	No	No	No	No	No
Patient population included in the antibiogram					
Inpatient	Yes	Yes	Yes	Yes	Yes
Emergency department	Yes	Yes	Yes	Yes	No
Outpatient	Yes	No	No	No	No
Urgent care	Yes	No	No	No	No
Only first isolate for a specific patient included in the antibiogram	Yes	No	Yes	No	Yes
Clindamycin results adjusted for inducible resistance testing					
*Staphylococcus* spp.	Yes	No	Yes	Yes	Yes
*Streptococcus pneumoniae*	Yes^a^	No	Yes	Yes	Yes
Use of post-2010 CLSI breakpoints for carbapenems for Enterobacterales	Yes	Yes	Yes	Yes	Yes
If yes, year post-2010 breakpoints adopted	2017	2019	2020	2020	2012
Testing for carbapenemase performed	Yes	Yes	Yes	Yes	No
If yes, method used for carbapenemase detection	BioFire, BCID, CarbaR	CarbaR	CarbaR	mCIM +/- eCIM	Specimens sent to state lab
Use of new CLSI breakpoints for cephalosporins for Enterobacterales	Yes	Yes	Yes	Yes	Yes
If yes, year new cephalosporin breakpoints adopted	2017	2019	2020	2020	2020
Testing for extended-spectrum beta-lactamases	Yes	Yes	Yes	Yes	Yes
Use of new CLSI breakpoints for fluoroquinolones for Enterobacterales	No^b^	Yes	Yes	Yes	Yes
If yes, year new fluoroquinolones breakpoints adopted for Enterobacterales	NA	2019	2020	2022	2022
Use of new CLSI breakpoints for fluoroquinolones for *Pseudomonas aeruginosa*	No^b^	Yes	Yes	Yes	Yes
If yes, year new fluoroquinolones breakpoints adopted for *P. aeruginosa*	NA	2019	2020	2022	2022
Lab performs respiratory cultures for patients with cystic fibrosis	Yes^c^	No	No	No	Yes
Use of updated CLSI breakpoints for piperacillin/tazobactam for Enterobacterales (2022) and *P. aeruginosa* (2023)	No	Yes^d^	No	No	No
Use of 2023 CLSI breakpoints for aminoglycosides for Enterobacterales and *P. aeruginosa*	No	No	No	No	No


Responses are based on 2021 hospital data and microbiology lab data.

GA is Georgia. ICU is intensive care unit. CLSI is Clinical and Laboratory Standards Institute. Spp. is species.

NA is not applicable.

a Adjustment for inducible clindamycin resistance for *S. pneumoniae* started in 2022.

b For Atlanta, the fluoroquinolone results are suppressed in the 2021-2023 antibiogram.

c For Atlanta, *P. aeruginosa* cystic fibrosis isolates are not included in the antibiogram.

d For Augusta, updated breakpoints for piperacillin/tazobactam were adopted in March 2023 for both Enterobacterales and *P. aeruginosa*.

The combined antibiotic susceptibility data for the five children’s hospitals in Georgia for 2023 are shown in Table 2. Clindamycin susceptibility differed between MSSA (78%) and MRSA (82%). The susceptibility of *S. pneumoniae* to amoxicillin (inferred from amoxicillin/clavulanate) was 96%. For *E. coli*, using the uncomplicated UTI breakpoints (≤16 µg/mL), susceptibility to cefazolin was 84%. Cefazolin susceptibility was 68% using the systemic infection breakpoint (≤2 µg/mL). For *E. coli* and *K. pneumoniae*, susceptibility to ceftriaxone was 92% and 90%, respectively. For all included gram-negative bacteria, susceptibility remained high to ciprofloxacin (90%–97%) and meropenem (95%–99%).

**Table 2.** Combined pediatric antibiotic susceptibility data for the 2023 year for the state of Georgia


	Ampicillin	Amox/clav	Oxacillin	Ceftriaxone(non-meningitic)	Ceftriaxone (meningitic)	Clindamycin^a^	TMP/SMX	Vancomycin	Linezolid	Levofloxacin	Nitrofurantoin (urine)				
**Gram-positive organisms**	Percent susceptible (number of isolates)														
MSSA			100 (1122)			78 (1113)	99 (1122)	100 (1122)							
MRSA			0 (566)			82 (565)	97 (566)	100 (566)							
*Enterococcus faecalis*	100 (254)							99 (254)	100 (209)		100 (249)				
*Streptococcus pneumoniae*		96 (215)		98 (241)	86 (224)	93 (243)		100 (243)		99 (220)					
	Ampicillin	Cefazolin (systemic)^b^	Ceftriaxone	Ceftazidime	Cefepime	Pip/tazo^d^	Meropenem	Gentamicin^d^	Tobramycin^d^	Amikacin^d^	TMP/SMX	Ciprofloxacin^c^	Levofloxacin^c^	Cefazolin (urine)	Nitrofurantoin (urine)
**Gram-negative organisms**	Percent susceptible (number of isolates)														
*Escherichia coli*	45 (2346)	68 (2251)	92 (2442)		93 (2442)	97 (2442)	99 (2442)	89 (645)		100 (549)	70 (2442)	90 (645)		84 (1892)	98 (2405)
*Klebsiella pneumoniae*		79 (444)	90 (495)		91 (495)	93 (495)	99 (495)	93 (150)	93 (150)	100 (99)	83 (495)	95 (150)			32 (425)
*Enterobacter cloacae* complex					94 (197)		99 (197)	100 (66)	98 (66)	100 (35)	87 (197)	97 (66)			
*Pseudomonas aeruginosa*				92 (403)	93 (464)	90 (464)	95 (464)		97 (100)	100 (39)		96 (111)	90 (50)		


MSSA is methicillin-susceptible *Staphylococcus aureus*. MRSA is methicillin-resistant *Staphylococcus aureus*. Amox/clav is amoxicillin/clavulanate. Pip/tazo is piperacillin/tazobactam. TMP/SMX is trimethoprim/sulfamethoxazole.

For a given bacterium, the differences in the total number of isolates are the result of differences in antibiotics included in susceptibility testing for the different hospitals.

a Augusta did not adjust for inducible clindamycin resistance for *S. aureus* for *S. pneumoniae*.

b Susceptibility results based on systemic breakpoints or when breakpoints used were not specified.

c Fluoroquinolone data for Atlanta were not included.

d Updated breakpoints for piperacillin/tazobactam and aminoglycosides were not adopted in 2023 except for Augusta, which began using updated piperacillin/tazobactam breakpoints in March of 2023.

**Figure 1.**
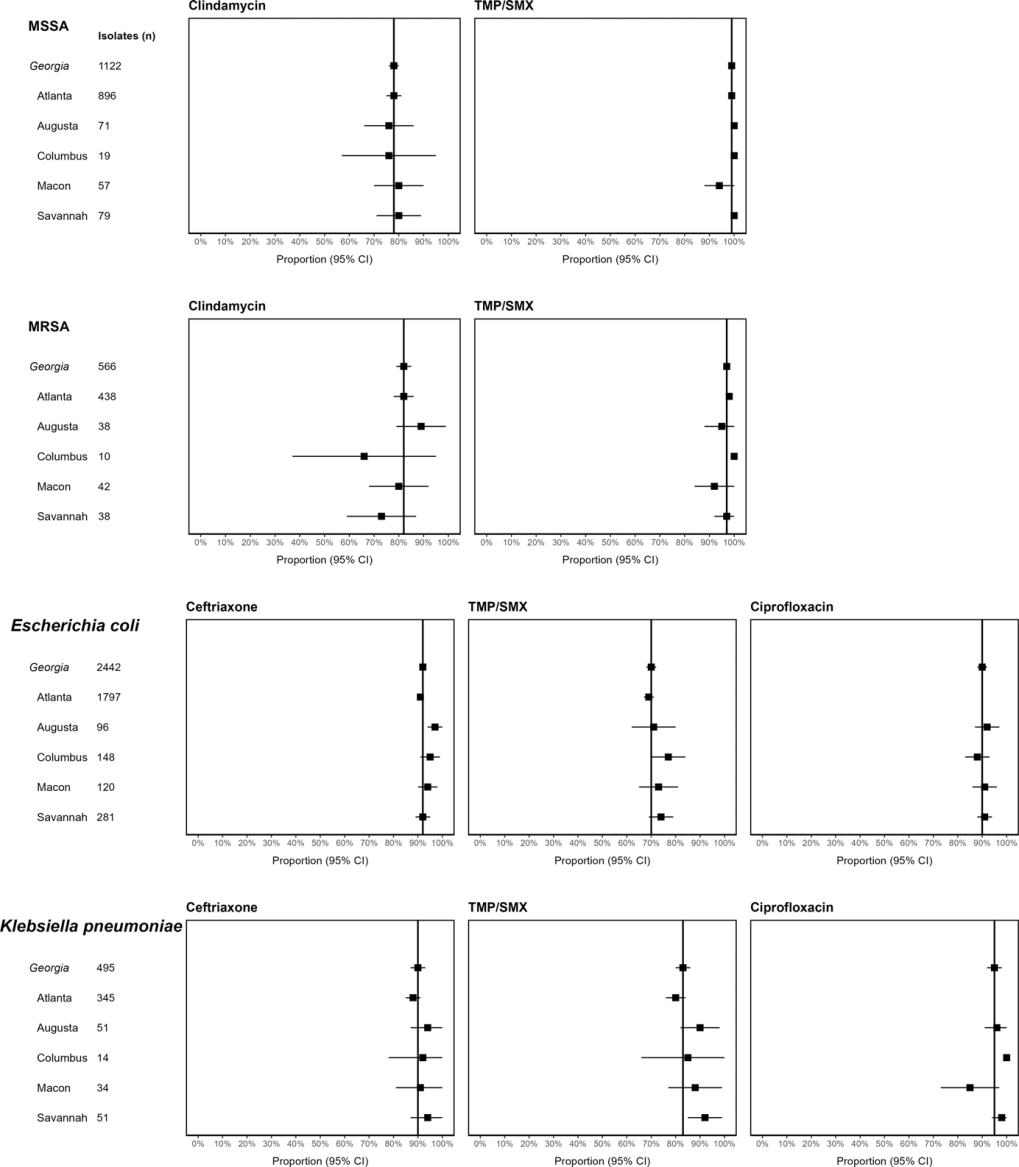
Comparison of Mean Antibiotic Susceptibilities for Selected Antibiotic-Pathogen Combinations from Pediatric Antibiograms for Individual Hospitals and Statewide Data in Georgia in 2023. Figure 1. A. MSSA. Figure 1. B. MRSA. Figure 1. C. *Escherichia coli.* Figure 1. D. *Klebsiella pneumoniae.* Forest plots represent mean and the 95% confidence interval. MSSA is methicillin-susceptible *Staphylococcus aureus*. MRSA is methicillin-resistant *Staphylococcus aureus*. TMP/SMX is trimethoprim/sulfamethoxazole. Ciprofloxacin data for Atlanta were not included. This decreased the number of Georgia isolates to 645 for *E. coli* and 150 for *K. pneumoniae*.

Figure 1 shows the comparison of individual hospital antibiotic susceptibility for MSSA, MRSA, *E. coli,* and *K. pneumoniae* with the statewide data for 2023. For MSSA for both clindamycin and trimethoprim/sulfamethoxazole (TMP/SMX), hospital rates were similar to the Georgia mean susceptibility, and CIs overlapped (Figure 1A). For MRSA, there was more variation in the hospital susceptibility rates to clindamycin and TMP/SMX, but the CIs overlapped with or were slightly greater than the Georgia mean (Figure 1B). For *E. coli*, hospital susceptibility rates were similar or slightly greater than the Georgia mean, and CIs overlapped for the included antibiotics. One exception was ceftriaxone susceptibility for Augusta in which the susceptibility was greater and the CI did not overlap with the Georgia data (Figure 1C). Similarly, for *K. pneumoniae*, hospital susceptibilities were similar or slightly greater than the Georgia mean and CIs overlapped. One exception was ciprofloxacin in Columbus, in which the susceptibility rate was greater, but the CIs did not overlap. Ciprofloxacin susceptibility in Macon was less than the Georgia mean but the CI overlapped (Figure 1D).

**Figure 2.**
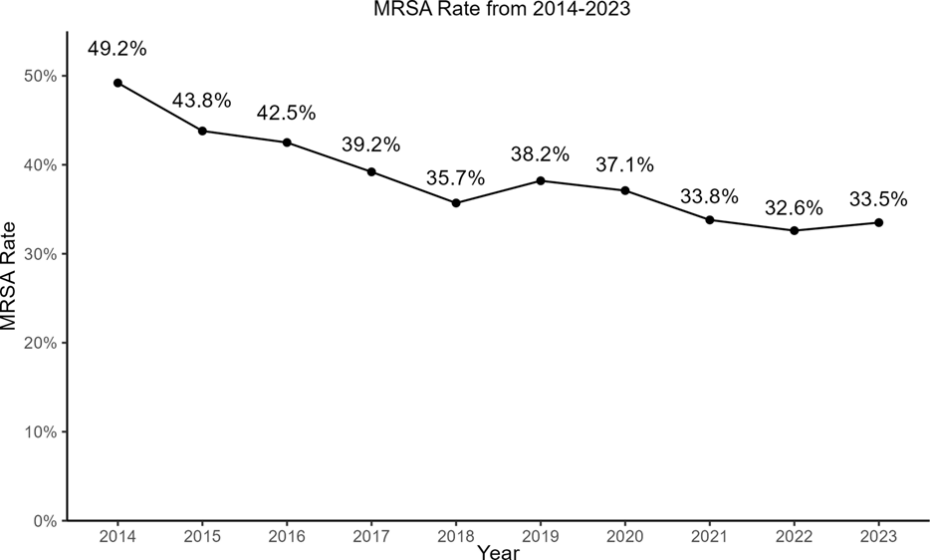
Change in the Rate of Methicillin-Resistant *Staphylococcus aureus* from Pediatric Isolates from 2014 through 2023 in Georgia. MRSA is methicillin-resistant *Staphylococcus aureus*.

**Figure 3.**
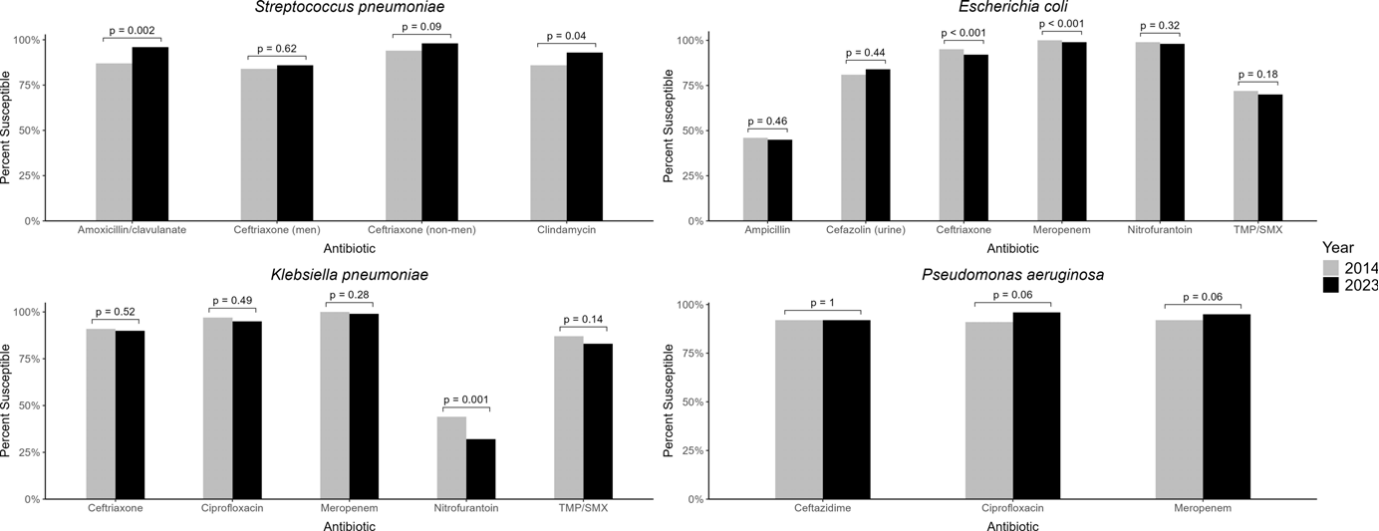
Comparison of Antibiotic Susceptibility Rates from Pediatric Isolates for Selected Bacteria between 2014 and 2023 in Georgia. Urine represents uncomplicated urinary tract infections breakpoints for cefazolin (≤16 µg/mL) for *E. coli.* Comparison is for 2017 and 2023 data. Men is meningitic. Non-men is non-meningitic. TMP/SMX is trimethoprim/sulfamethoxazole.

The MRSA rate significantly decreased from 49% in 2014 to 33.5% in 2023 (*P* < 0.001) (Figure 2). Susceptibility to clindamycin remained stable for MSSA (78%) and MRSA (82%). Combined antibiogram data were used to show changes in susceptibility between 2014 and 2023 (Figure 3). *S. pneumoniae* showed significantly increased susceptibility to amoxicillin (inferred from amoxicillin/clavulanate) from 87% to 96% (*P* = 0.002), and to clindamycin from 86% to 93% (*P* = 0.04) between 2014 and 2023. For *E. coli,* there was a significant decrease in susceptibility from 2014 to 2023 for cefazolin (90% to 84%) (UTI breakpoints), ceftriaxone (95% to 92%) and meropenem (100% to 99%). For *K. pneumoniae*, there was a significant decrease in susceptibility to nitrofurantoin from 47% to 32%. For *P. aeruginosa*, there was a nonsignificant increase in susceptibility for ciprofloxacin and meropenem.

## Discussion

Many pediatric healthcare facilities struggle with having enough isolates to create antibiograms unimpacted by sampling bias, but as the threat of antibiotic resistance continues, accurate antibiograms are essential to guide antibiotic prescribing in children. We combined antibiogram data from the five children’s hospitals in Georgia, each hospital located in a different part of the state, to create the first pediatric-specific antibiogram for the state. By creating statewide antibiograms from 2014 through 2023, we were able to identify changes in antibiotic susceptibility over the past 10 years in Georgia.

Three of the five children’s hospitals contributing antibiogram data in this project frequently had fewer than the CLSI-recommended 30 isolates for bacteria included in their annual antibiograms. In a pooled nationwide pediatric antibiogram, only 16% of children’s hospitals reported requiring a minimum of 30 isolates when creating their local antibiograms suggesting that this is a frequent challenge for pediatric hospitals.^10^ When fewer than the recommended number of isolates are used to create a local antibiogram, it can be difficult to interpret whether the reported antibiotic susceptibility is representative of actual antibiotic susceptibility. Regional antibiograms, which combine isolates from multiple hospitals or groups, can overcome this problem and have been successfully created in several states; although, these have primarily included adult data.^7,9,11,13^ Significant regional differences or a single hospital providing the majority of isolates could impact whether combined data are representative of the local susceptibility. When comparing interhospital variation, combined regional antibiograms have demonstrated that over 90% of hospital susceptibility data clustered around the regional mean.^7,9^ The Atlanta children’s healthcare system contributed over 75% of the isolates to the statewide antibiogram. We compared the antibiotic susceptibility data of each individual hospital with the statewide mean to determine whether there were significant differences. Local antibiotic susceptibility was similar to or slightly greater than the statewide mean in most comparisons, and local confidence intervals overlapped the statewide mean in nearly all comparisons. Although data from Atlanta slightly reduced the Georgia susceptibility data, the statewide data remained representative of local antibiotic susceptibility.

Using pediatric-specific antibiograms submitted from 55 US hospitals from 2005 through 2011, a combined pediatric antibiogram was created, providing a snapshot of antibiotic susceptibility in children in the US.^10^ In 2010–11, MRSA represented about 50% of *S. aureus* isolates with 79% of *S. aureus* isolates were susceptible to clindamycin. For *E. coli* and *K. pneumoniae*, susceptibility to ceftriaxone (94%–96%) and meropenem (98%–100%) were high. In 2014, our Georgia antibiotic susceptibility data were similar to the nationwide antibiotic susceptibility data for *S. aureus* and gram-negative bacilli reported in 2011. Overall, antibiotic susceptibility reported in our antibiogram remained stable for the past ten years, but there were a few notable changes. The rate of MRSA decreased significantly, now representing only about a third of *S. aureus* isolates. This is consistent with national trends showing decreased rates of MRSA.^14–16^ We also identified significantly increased *S. pneumoniae* susceptibility to amoxicillin and clindamycin. Studies have shown reduction in antibiotic-resistant serotype 19A after the 2010 introduction of the pneumococcal conjugate vaccine 13; however, the reported change in antibiotic susceptibility varied.^17,18^ In 2014, *S. pneumoniae* antibiotic susceptibility in Georgia was comparable to the antibiotic susceptibility reported in these studies. Our data show further improvement in pneumococcal antibiotic susceptibility, but it is unclear the relative impact ongoing serotype changes and antibiotic stewardship efforts have had. Between 2014 and 2023, *E. coli* and *K. pneumoniae* susceptibility remained stable but decreased slightly. In a large multicenter cohort study of neonates with *E. coli* infections from 2009 through 2017, antibiotic susceptibility was similar to Georgia susceptibility data and remained stable over time. There was a nonsignificant gradual increase in *E. coli* with an extended-spectrum beta-lactamase phenotype.^19^ Our data identified small but statistically significant decreases in *E. coli* susceptibility to ceftriaxone and meropenem over the past ten years. These data coupled with prior nationwide data are concerning for a slow-moving but worrisome trend. Although our data is limited to pediatric antibiotic susceptibility in Georgia, it has been consistent with previously published US data and provides additional information regarding changes in pediatric antibiotic susceptibility over the past ten years.^10,14,15,17–19^

One of the most practical uses of an antibiogram is to provide susceptibility data that can be used to reinforce treatment guidelines A recent systematic review showed that including antibiograms as part of multifaceted stewardship interventions may improve antibiotic use and appropriateness.^20^ The majority of antibiotics prescribed to children occur in the outpatient setting.^21^ Unfortunately, most community pediatricians either do not have access to antibiogram data or are unsure whether a hospital’s antibiogram applies to their patient population.^22^ Adult data comparing antibiotic susceptibility of outpatient and inpatient isolates demonstrate similar susceptibility for some antibiotic-pathogen combinations and greater outpatient susceptibility for other antibiotic-pathogen combinations.^23^ Regional or statewide pediatric antibiograms are needed and can provide community pediatricians with additional prescribing information, but additional studies are needed to identify whether there are important differences between pediatric inpatient and outpatient antibiotic susceptibility.

Evidence-based treatment guidelines have been published, which recommend amoxicillin as the first-line empiric antibiotic treatment for acute respiratory tract infections, of which *S. pneumoniae* is a major cause.^24–26^. Continued improvement of *S. pneumoniae* susceptibility in isolates from children in Georgia support the use of amoxicillin for upper and lower respiratory tract infections. The majority of soft tissue abscesses are caused by *S. aureus*. In conjunction with abscess drainage, Georgia antibiogram data support the use of TMP/SMX for treatment, but due to lower clindamycin susceptibility (78%–82%), it should be prescribed more cautiously. *E. coli* is the most common cause of UTIs in children, representing at least 80% of infections.^27^ First-generation cephalosporins are recommended for empiric treatment of UTIs in children, but if local resistance patterns show increased resistance (example ≥ 15%), local antibiograms should be used to guide antibiotic treatment.^28^ Over the past couple years, *E. coli* susceptibility to first-generation cephalosporins in Georgia has decreased to 84%–85%, using urine-specific breakpoints.^28^ Therefore, clinicians should incorporate local susceptibility data when choosing empiric antibiotics for UTIs. Altogether, antibiotic susceptibility patterns in Georgia are consistent with current evidence-based antibiotic treatment recommendations for common pediatric infections.

The results of this pediatric antibiogram are subject to a few notable limitations. First, the isolates used to create the different hospital antibiograms utilized specimens obtained primarily in the emergency departments and inpatient settings. This may have skewed results towards lower susceptibility compared to what may be seen in the community at large. Second, Atlanta contributed the greatest number of isolates for all included bacteria potentially overrepresenting the statewide susceptibility; however, the antibiotic susceptibility confidence intervals for the other hospitals overlapped the statewide mean. Hospitals adopted the updated CLSI interpretive criteria for cephalosporins, carbapenems, and fluoroquinolones at different times. These differences may have impacted the evaluation of susceptibility trends over time, with some of the decrease in observed susceptibility over time, in part, due to changes in interpretive criteria.

Despite the majority of isolates coming from a single large hospital and some differences in the timing various breakpoints were adopted, we were able to create a representative statewide pediatric antibiogram. Our data are consistent with previously reported US susceptibility data in children and provide additional information on changes in antibiotic resistance over the past ten years, including improved antibiotic susceptibility for gram-positive bacteria and stable but slightly decreased antibiotic susceptibility for gram-negative bacilli. Future work will include linking the statewide antibiogram data to evidence-based treatment guidelines for common pediatric infections and disseminating this information to pediatricians and evaluating the impact on their prescribing behaviors.

## Supporting information

Linam et al. supplementary material Linam et al. supplementary material

## Supplementary material

For supplementary material accompanying this paper visit http://doi.org/10.1017/ash.2025.32. click here to view supplementary material

## Abbreviations:

MSSA: methicillin-susceptible *Staphylococcus aureus*

MRSA: methicillin-resistant *Staphylococcus aureus*

CLSI: Clinical and Laboratory Standards Institute

UTI: Urinary tract infection

TMP/SMX: Trimethoprim/sulfamethoxazole

CI: Confidence interval

## Article summary:

A statewide pediatric antibiogram in Georgia shows decreased MRSA and improved pneumococcal susceptibility but decreased gram-negative bacilli susceptibility over the past ten years.

## References

[1] Dellit TH , Owens RC , McGowan JE , Jr., et al. Infectious diseases society of America and the society for healthcare epidemiology of America guidelines for developing an institutional program to enhance antimicrobial stewardship. Clin Infect Dis 2007;44:159–177. 17173212 10.1086/510393

[2] Core Elements of Hospital Antibiotic Stewardship Programs. Centers for Disease Control and Prevention website. 2019; https://www.cdc.gov/antibiotic-use/core-elements/hospital.html. Accessed November 1, 2023.

[3] Cairns KA , Doyle JS , Trevillyan JM , et al. The impact of a multidisciplinary antimicrobial stewardship team on the timeliness of antimicrobial therapy in patients with positive blood cultures: a randomized controlled trial. J Antimicrob Chemother 2016;71:3276–3283. 27494917 10.1093/jac/dkw285

[4] Truong WR , Hidayat L , Bolaris MA , Nguyen L , Yamaki J . The antibiogram: key considerations for its development and utilization. JAC Antimicrob Resist 2021;3:dlab060. 10.1093/jacamr/dlab060 PMC8210055 34223122

[5] Clinical and Laboratory Standards Institute (CLSI). Approved guideline M39. In: Analysis and Presentation of Cumulative Antimicrobial Susceptibility Test Data. 5th edition ed. Wayne, PA: CLSI; 2022.

[6] Moehring RW , Hazen KC , Hawkins MR , Drew RH , Sexton DJ , Anderson DJ . Challenges in preparation of cumulative antibiogram reports for community hospitals. J Clin Microbiol 2015;53:2977–2982. 26179303 10.1128/JCM.01077-15 PMC4540907

[7] Butler DA , Biagi M , Gupta V , et al. Development of a 51-hospital Chicagoland regional antibiogram and comparison to local hospital and national surveillance data. Infect Control Hosp Epidemiol 2020;41:1409–1418. 32886058 10.1017/ice.2020.334

[8] Fridkin SK , Pack J , Licitra G , et al. Creating reasonable antibiograms for antibiotic stewardship programs in nursing homes: analysis of 260 facilities in a large geographic region, 2016-2017. Infect Control Hosp Epidemiol 2019;40:839–846. 31133088 10.1017/ice.2019.112

[9] Hostler CJ , Moehring RW , Ashley ESD , et al. Feasibility and value of developing a regional antibiogram for community hospitals. Infect Control Hosp Epidemiol 2018;39:718–722. 29681253 10.1017/ice.2018.71 PMC6664445

[10] Tamma PD , Robinson GL , Gerber JS , et al. Pediatric antimicrobial susceptibility trends across the United States. Infect Control Hosp Epidemiol 2013;34:1244–1251. 24225608 10.1086/673974

[11] Var SK , Hadi R , Khardori NM . Evaluation of regional antibiograms to monitor antimicrobial resistance in Hampton Roads, Virginia. Ann Clin Microbiol Antimicrob 2015;14:22. 25890362 10.1186/s12941-015-0080-6 PMC4397712

[12] Fridkin SK . Increasing prevalence of antimicrobial resistance in intensive care units. Crit Care Med 2001;29:N64–68. 10.1097/00003246-200104001-00002 11292878

[13] Plante JG , Winders HR , Bookstaver PB , et al. Regional and statewide antibiograms as targeted interventions against antibiotic resistance. Infect Control Hosp Epidemiol 2021;42:503–505. 32583760 10.1017/ice.2020.273

[14] Spaulding AB , Thurm C , Courter JD , et al. Epidemiology of Staphylococcus aureus infections in patients admitted to freestanding pediatric hospitals, 2009-2016. Infect Control Hosp Epidemiol 2018;39:1487–1490. 30370879 10.1017/ice.2018.259

[15] Stultz JS , Benefield E , Lee KR , Bashqoy F , Pakyz AL . A multicenter analysis of changes in pediatric antibiotic susceptibilities among Staphylococcus aureus and Pseudomonas aeruginosa isolates: 2014-2018. J Pediatr Pharmacol Ther 2022;27:330–339. 35558344 10.5863/1551-6776-27.4.330 PMC9088438

[16] National Infection & Death Estimates for Antimicrobial Resistance. Center for Disease Control and Prevention website. 2021; https://www.cdc.gov/drugresistance/national-estimates.html. Accessed November 1, 2023.

[17] Desai AP , Sharma D , Crispell EK , et al. Decline in pneumococcal nasopharyngeal carriage of vaccine serotypes after the introduction of the 13-valent pneumococcal conjugate vaccine in children in Atlanta, Georgia. Pediatr Infect Dis J 2015;34:1168–1174. 26226445 10.1097/INF.0000000000000849

[18] Lee GM , Kleinman K , Pelton S , et al. Immunization, antibiotic use, and pneumococcal colonization over a 15-year period. Pediatrics 2017;140:e20170001. 28978716 10.1542/peds.2017-0001 PMC5654389

[19] Flannery DD , Akinboyo IC , Mukhopadhyay S , et al. Antibiotic susceptibility of Escherichia coli among infants admitted to neonatal intensive care units across the US from 2009 to 2017. JAMA Pediatr 2021;175:168–175. 33165599 10.1001/jamapediatrics.2020.4719 PMC7653538

[20] Khatri D , Freeman C , Falconer N , de Camargo Catapan S , Gray LC , Paterson DL. Clinical impact of antibiograms as an intervention to optimize antimicrobial prescribing and patient outcomes-A systematic review. Am J Infect Control 2023;52:107–122. 37604208 10.1016/j.ajic.2023.08.013

[21] Chai G , Governale L , McMahon AW , Trinidad JP , Staffa J , Murphy D . Trends of outpatient prescription drug utilization in US children, 2002–2010. Pediatrics 2012;130:23–31. 22711728 10.1542/peds.2011-2879

[22] Spiekerman KM , Patel SJ , Patel R , Kociolek LK . Availability, perceptions, and characteristics of antibiograms among Illinois pediatricians. Infect Drug Resist 2016;9:269–274. 27980428 10.2147/IDR.S122879 PMC5147404

[23] McGregor JC , Bearden DT , Townes JM , et al. Comparison of antibiograms developed for inpatients and primary care outpatients. Diagn Microbiol Infect Dis 2013;76:73–79. 23541690 10.1016/j.diagmicrobio.2013.01.026 PMC3658613

[24] American Academy of Pediatrics Subcommittee on Management of Acute Otitis M. Diagnosis and management of acute otitis media. Pediatrics 2004;113:1451–1465. 15121972 10.1542/peds.113.5.1451

[25] Wald ER , Applegate KE , Bordley C , et al. Clinical practice guideline for the diagnosis and management of acute bacterial sinusitis in children aged 1 to 18 years. Pediatrics 2013;132:e262–280. 23796742 10.1542/peds.2013-1071

[26] Bradley JS , Byington CL , Shah SS , et al. The management of community-acquired pneumonia in infants and children older than 3 months of age: clinical practice guidelines by the pediatric infectious diseases society and the infectious diseases society of America. Clin Infect Dis 2011;53:e25–76. 21880587 10.1093/cid/cir531 PMC7107838

[27] Edlin RS , Shapiro DJ , Hersh AL , Copp HL . Antibiotic resistance patterns of outpatient pediatric urinary tract infections. J Urol 2013;190:222–227. 23369720 10.1016/j.juro.2013.01.069 PMC4165642

[28] Mattoo TK , Shaikh N , Nelson CP . Contemporary management of urinary tract infection in children. Pediatrics 2021;147:e2020012138. 33479164 10.1542/peds.2020-012138

