# Supplementary material for: Development of a multi-year pediatric antibiogram in Georgia identifies antibiotic resistance changes over the past ten years
Source: Antimicrob Steward Healthc Epidemiol. 2025 Feb 12;5(1):e37. doi: 10.1017/ash.2025.32 (PMC11822576; doi:10.1017/ash.2025.32)
Supplement: Linam et al. supplementary material [file S2732494X25000324sup001.docx]

| **Supplemental Table. Bacteria and Antibiotics Included in the Statewide Georgia Pediatric Antibiogram** | | | | | | | | | | | | | | | |
| --- | --- | --- | --- | --- | --- | --- | --- | --- | --- | --- | --- | --- | --- | --- | --- |
|  | Ampicillin | Amox/clav | Oxacillin | Ceftriaxone (non-meningitic) | Ceftriaxone  (meningitic) | Clindamycin | TMP/SMX | Vancomycin | Levofloxacin | Nitrofurantoin  (urine) |  |  |  |  |  |
| **Gram positive organisms** |  |  |  |  |  |  |  |  |  |  |  |  |  |  |  |
| MSSA |  |  | x |  |  | x | x | x |  |  |  |  |  |  |  |
| MRSA |  |  | x |  |  | x | x | x |  |  |  |  |  |  |  |
| *Enterococcus faecalis* | x |  |  |  |  |  |  | x |  | x |  |  |  |  |  |
| *Streptococcus pneumoniae* | x | x |  | x | x | x |  | x | x |  |  |  |  |  |  |
|  | Ampicillin | Cefazolin | Ceftriaxone | Ceftazidime | Cefepime | Pip/tazo | Meropenem | Gentamicin | Tobramycin | Amikacin | TMP/SMX | Ciprofloxacin | Levofloxacin | Cefazolin  (urine) | Nitrofurantoin  (urine) |
| **Gram negative organisms** |  |  |  |  |  |  |  |  |  |  |  |  |  |  |  |
| *Escherichia coli* | x | x | x |  | x | x | x | x |  | x | x | x |  | x | x |
| *Klebsiella pneumoniae* |  | x | x |  | x | x | x | x | x | x | x | x |  |  | x |
| *Enterobacter cloacae* complex |  |  |  |  | x |  | x | x | x | x | x | x |  |  | x |
| *Pseudomonas aeruginosa* |  |  |  | x | x | x | x |  | x | x |  | x | x |  |  |
| MSSA is methicillin-susceptible *Staphylococcus aureus*. MRSA is methicillin-resistant *S. aureus*. Amox/clav is amoxicillin/clavulanate. Pip/tazo is piperacillin/tazobactam. TMP/SMX is trimethoprim/sulfamethoxazole. | | | | | | | | | | | | | | | |
